# Air quality indicators: when data disappear

**DOI:** 10.2471/BLT.25.293937

**Published:** 2025-06-01

**Authors:** Ankita S Achanta, Ther W Aung

**Affiliations:** a School of Medicine, Case Western Reserve University, 10900 Euclid Ave, Cleveland, Ohio, 44106, United States of America.

We recently assessed the health impact of improved ambient air quality in five Indian megacities following the movement restrictions during the coronavirus disease 2019 pandemic. We used (at the time) publicly available particulate matter data collected by embassies and consulates of the United States of America. Our paper was accepted for publication in the *Bulletin of the World Health Organization*; however, before the article’s publication, in March 2025, public access to this data set was suspended.^1^ Because the raw data were no longer publicly accessible, the *Bulletin* withdrew its acceptance of the paper. The removal of publicly available air quality data will have negative consequences for global research and for the health of billions of people in low- and middle-income countries.

Since 2008, the United States Department of State has collected air pollution data at its embassies and consulates and made them publicly available, providing a reliable source of real-time air quality information in many countries. Such access has had many positive spillover effects, including advances in scientific research, environmental health awareness and changes in policies and practices in low- and middle-income countries. For example, air quality data from the State Department have been used to conduct comparative studies in India,^2^^,^^3^ China^4^^,^^5^ and across continents.^6^ Researchers have also used the data to identify potential sources of particulate matter,^6^ a key component of air pollution that is hazardous to health. Knowledge of these sources is critical for developing air quality management plans and therefore reducing its health impacts.

Furthermore, many governments in low- and middle-income countries may not have the financial and technical resources to install and maintain high-quality air monitors. Such monitors can carry an annual operational cost of over 130 000 United States dollars per monitor.^7^ The State Department air monitoring has partially filled this critical information gap by collecting air quality data in over 40 cities^6^ globally and by making the data freely and publicly available. For example, in India, each of the five megacities included in our assessment – home to over 10 million people per city – have high-quality air monitors that have provided data since 2017. Although one monitor in a city cannot provide representative exposure data, particularly in densely populated cities, it can shed light on spatial differences between cities and temporal trends, such as hourly, daily or annual changes in air pollution levels. While other sources of publicly available air quality data exist, such as satellite remote sensing and a growing number of low-cost sensors, ground-based high-quality monitors are a critical part of an air quality data ecosystem, because researchers need a combination of data sources to build, validate and refine exposure models.^7^

Exposure to high levels of fine particulate matter is associated with a range of adverse health outcomes including premature death, cancer, respiratory and cardiovascular diseases, and increased child mortality.^8^ While the evidence of the health impacts is robust, much of this evidence is from studies of populations in high-income countries. The State Department’s air quality data have enabled researchers to build predictive models^9^^,^^10^ and to analyse the connection between air pollution and health^10^ in countries where such research would be impossible on individual research project budgets.

The State Department’s air quality data has also been instrumental in changing policy and practice. A widely known example is in China, where social media tweets on air quality from the United States Embassy in 2010 led to the Chinese government proposing new air quality standards.^11^ Additionally, in countries with United States embassy monitors, particulate matter has declined, resulting in decreased risk of premature mortality.^11^

The removal of public access to air quality data from the State Department will undermine research in countries where data are scarce and a large proportion of the population is exposed to hazardous levels of air pollution. Governments that lack the means to generate their own air quality data will be excluded from an important resource, and without that knowledge, citizens will be unable to participate in environmental health actions. Until all governments invest in their own air quality monitoring solutions, the air monitoring infrastructure already in place at United States embassies remains a valuable resource. Given the substantial investment made and the minimal resources required to continue data sharing, restoring public access to this information would be an effective step towards strengthening health-protective policies and practices against air pollution. 
